# Tofacitinib Loaded Squalenyl Nanoparticles for Targeted Follicular Delivery in Inflammatory Skin Diseases

**DOI:** 10.3390/pharmaceutics12121131

**Published:** 2020-11-24

**Authors:** Rebekka Christmann, Duy-Khiet Ho, Jenny Wilzopolski, Sangeun Lee, Marcus Koch, Brigitta Loretz, Thomas Vogt, Wolfgang Bäumer, Ulrich F. Schaefer, Claus-Michael Lehr

**Affiliations:** 1 Helmholtz-Institute for Pharmaceutical Research Saarland (HIPS)—Helmholtz Centre for Infection Research (HZI), 66123 Saarbrücken, Germany; Rebekka.Christmann@helmholtz-hips.de (R.C.); DuyKhiet.Ho@helmholtz-hips.de (D.-K.H.); Sangeun.Lee@helmholtz-hips.de (S.L.); Brigitta.Loretz@helmholtz-hips.de (B.L.); 2 Department of Pharmacy, Saarland University, 66123 Saarbrücken, Germany; ufs@mx.uni-saarland.de; 3 Institute of Pharmacology and Toxicology, Department of Veterinary Medicine, Freie Universität Berlin, 14195 Berlin, Germany; Jenny.Wilzopolski@fu-berlin.de (J.W.); Wolfgang.Baeumer@fu-berlin.de (W.B.); 4 INM-Leibniz Institute for New Materials, 66123 Saarbrücken, Germany; Marcus.Koch@leibniz-inm.de; 5 Department of Dermatology, Saarland University Hospital, 66421 Homburg/Saar, Germany; Thomas.Vogt@uks.eu

**Keywords:** targeted drug delivery, hair follicle, *in vivo* allergic dermatitis mouse model, follicular delivery, interfollicular delivery, nanoparticles, squalene

## Abstract

Tofacitinib (TFB), a Janus kinase inhibitor, has shown excellent success off-label in treating various dermatological diseases, especially alopecia areata (AA). However, TFB’s safe and targeted delivery into hair follicles (HFs) is highly desirable due to its systemic adverse effects. Nanoparticles (NPs) can enhance targeted follicular drug delivery and minimize interfollicular permeation and thereby reduce systemic drug exposure. In this study, we report a facile method to assemble the stable and uniform 240 nm TFB loaded squalenyl derivative (SqD) nanoparticles (TFB SqD NPs) in aqueous solution, which allowed an excellent loading capacity (LC) of 20%. The SqD NPs showed an enhanced TFB delivery into HFs compared to the aqueous formulations of plain drug in an *ex vivo* pig ear model. Furthermore, the therapeutic efficacy of the TFB SqD NPs was studied in a mouse model of allergic dermatitis by ear swelling reduction and compared to TFB dissolved in a non-aqueous mixture of acetone and DMSO (7:1 *v*/*v*). Whereas such formulation would not be acceptable for use in the clinic, the TFB SqD NPs dispersed in water illustrated a better reduction in inflammatory effects than plain TFB’s aqueous formulation, implying both encouraging good *in vivo* efficacy and safety. These findings support the potential of TFB SqD NPs for developing a long-term topical therapy of AA.

## 1. Introduction

Alopecia areata (AA) is one of the major non-scarring hair loss disorders with a lifetime incidence of 2% [1,2,3]. AA is a complex disorder that involves breakdown of the immune privilege of anagen hair follicles (HFs), leads to an attack of the immune system, and causes reversible hair loss with a preserved HF [1,4]. Although AA is a non-life-threatening disease, it still has a significant effect on the patients’ quality of life [1,5]. The most common off-labeled treatment regimens for AA include corticosteroids—applied intralesionally, topically or systemically, topical minoxidil, oral methotrexate, or topical immunotherapy [6], while an approved medicine is not available due to the missing therapeutic outcome prediction and the often-seen relapses [6].

A recently developed Janus kinase inhibitor (JAK-inhibitor) has demonstrated an impressive hair regrowth in long-term alopecia universalis [7,8]. Namely, tofacitinib (TFB)—an FDA-approved immunosuppressant against rheumatoid arthritis, psoriasis arthritis, and ulcerative colitis [9], which inhibits JAK 1 and 3, has shown promising treatment progress on human and mice [10,11,12,13]. However, continuous treatment is necessary to gain a possible persistent success in hair regrowth [12,13]. Unfortunately, long-term systemic treatment with TFB can cause a broad and severe spectrum of adverse effects due to the wide bioactive range of such JAKs [14]. JAKs are part of the intracellular signaling pathway transducing cytokine signaling of both the types I and II cytokine receptor family. Thus, JAK/STAT (signal transducers and activators of transcription) dependent cytokines have a broad spectrum of action in humans, especially in hematopoiesis, inflammation induction, and immune response control [14,15]. In 2019, TFB received an FDA-boxed warning due to an “increased risk of blood clots and death with higher dose” [16]. Those facts emphasize the necessity of generating a safer treatment profile for TFB, especially in non-life-threatening diseases like AA [5,17].

The TFB targeted HF delivery is considered as a good strategy to reduce the high systemic drug levels and adverse effects. The topical application of the nanoparticulate drug delivery system (NDDS) to the skin is able to (i) enhance drug solubility [18], (ii) deliver drugs targeted into HFs [19,20], (iii) build up a drug depot [20,21], and (iv) release the drug in a controlled manner [22,23]. Consequently, nanoparticles (NPs) can deliver a higher amount of drug to HFs compared to plain drug solution—meaning to maximize the follicular penetration while minimizing the interfollicular permeation of drug molecules [22,23,24]. The primary mechanism of NPs deposition in HFs is the postulated ratchet effect of cuticle cells in the hair shaft [20,25]. However, we have previously demonstrated the deposition of NPs also in AA affected hairless human scalp HFs [26].

NDDS excipients composition can be chosen depending on their purpose, beside the conventional approach of biodegradable polymers, for example, metal-based NDDS are used as antimicrobial agents [27]. In addition, NDDS can release their cargo upon a triggering stimulus, e.g., by ultrasound or infrared A radiation [28,29,30].

Taking advantage of an NP approach, we aimed to develop a NDDS for TFB targeted delivery to AA affected HFs. Importantly, since a high dose of TFB—up to 5 or 10 mg orally twice daily—is clinically required [9], a NDDS for TFB must have a high drug loading capacity (LC). Moreover, such a NDDS should also be safe and easily prepared to meet future translational requirements [31]. The conventional NDDS’s based on biodegradable polymers (e.g., poly (lactic-co-glycolic acid) (PLGA)) or nanostructured lipid carriers (NLCs) offer good biocompatibility and are facile to produce. LC had, however, been reported to be lower than 5% (*w*/*w*) [32,33], which was in good agreement with our attempts, although previously a study claimed around 30% LC for TFB citrate salt loaded PLGA particles [34].

Squalene alone accounts for around 12% of the human sebum’s major components [35] and is a precursor of the cholesterol biosynthesis throughout the human body [35,36]. Interestingly, squalene is not further transformed in the sebaceous cells, which makes the skin the richest squalene containing tissue in the human body [35,36]. Thus, squalene-based NPs for targeted follicular delivery would be an excellent choice. Squalenoylation technology, which consists of linking a hydrophobic squalenyl moiety to a more hydrophilic drug molecule, can significantly improve drug LC in self-assembled squalenoylated NPs. This, moreover, could enhance the therapeutic efficacy of the drugs [37,38]. Unfortunately, the secondary amine in the pyrrole ring, which is the only availably reactive group in TFB, is a relatively weak nucleophile for covalently coupling reactions. Possible attempts to synthesize TFB prodrug are not facile [39]. Only the successful formation of a carbamate linkage was reported, leading to undesired long cleavage times for over three weeks [39]. Moreover, TFB is degraded in harsh conditions making it less favorable in the chemically squalenyl modification [39,40,41].

Recently, we have reported an alternative to the squalenoylation approach, in which we used amphiphilic squalenyl derivatives to formulate the surfactant-free self-assembled NPs allowing significantly high drug LC in both hydrophilic and hydrophobic compartments [42,43,44].

Based on these studies we employed an anionic squalenyl derivative (SqD)—squalenyl hydrogen sulfate—to produce the TFB loaded SqD NPs (TFB SqD NPs). This SqD is composed of a hydrophilic hydrogen sulfate head-group covalently bonded to a hydrophobic moiety. We speculated that SqD NPs could potentially allow the loading of TFB via charged interaction in the NPs shell and hydrophobic interaction in the NPs core, hence giving a high LC of TFB. The TFB SqD NPs were moreover expected to provide an enhanced TFB delivery in HFs and therapeutic effects.

We here first describe the preparation and characterization of TFB SqD NPs. In the subsequent studies, we investigated the performance of TFB SqD NPs compared to formulations of the plain drug and vehicle controls in some established *ex vivo* and *in vivo* models. The *ex vivo* pig ear model was adopted from previously reported ones [45,46] and used to determine the TFB penetration and permeation pathways—into either follicular or interfollicular skin areas. A well-established *in vivo* allergic dermatitis mouse model was, in turn, used to evaluate the therapeutic efficacy of the formulations in reducing an inflammatory response, which corresponds to a reduction in the ear swelling upon being challenged with allergen [47]. Our *ex vivo* and *in vivo* findings reveal the TFB SqD NPs as safe and efficient for targeted drug delivery to the hair follicles after topical administration.

## 2. Materials and Methods

### 2.1. Materials

TFB free base (TFB) and TFB citrate salt (TFB-C) were purchased from LC Laboratories, Massachusetts, US. TFB-C was manufactured to enhance the water solubility of TFB and was in this study used to prepare an aqueous drug reference solution. SqD was synthesized as described previously [43,44]. Ammonium acetate (LC-MS grade) was purchased from Sigma-Aldrich, Munich, Germany. Disodium hydrogen phosphate heptahydrate and sodium dihydrogen phosphate hydrate were purchased from Carl Roth, Karlsruhe, Germany. Tetrahydrofuran (HPLC grade) (THF), ethanol, absolute (HPLC grade; EtOH), formic acid (LC-MS grade), and dimethyl sulfoxide (DMSO) were purchased from Fisher Scientific, Leicestershire, UK. Acetone (HPLC grade) was purchased from Honeywell Riedel-deHäen, Fisher Scientific GmbH, Schwerte, Germany. Methanol (MeOH) was obtained from VWR Chemicals, Darmstadt, Germany. A disposable biopsy punch with plunger with a 1 mm diameter was obtained from kai europe GmbH, Solingen, Germany. Millex^®^-LG syringe driven filter units (PTFE, 0.20 μm) were purchased from Merck Millipore, Darmstadt, Germany. Faber Castell^®^ MULTIMARK permanent 152399, 0.4 mm in black was purchased from A.W. Faber-Castell Vertrieb GmbH, Stein, Germany. Tesafilm kristallklar 33 m × 19 mm was a gift from tesa SE, Hamburg, Germany. Dialysis bag SpetraPor^®^7, MWCO 10 kDa, consisting of regenerative cellulose was purchased from Repligen Corporation, California, US. Milli-Q water purification system from Merck Millipore (Darmstadt, Germany) was used to prepare purified water (water).

### 2.2. Animal Procedure and Ethics Statement

The animal experiment was approved on 27th of November in 2017 by state agency (LAGeSo Berlin, approval number: G 0234/17). The experiments were conducted in female BALB/c (BALB/cAnNCrl) aged 6–8 weeks. Mice were obtained from Charles River Laboratories (Sulzfeld, Germany). Mice received a standard diet and water ad libitum. After arrival, mice were acclimatized to their environment for 1 week.

### 2.3. Preparation of TFB SqD NPs

**Figure 1:**
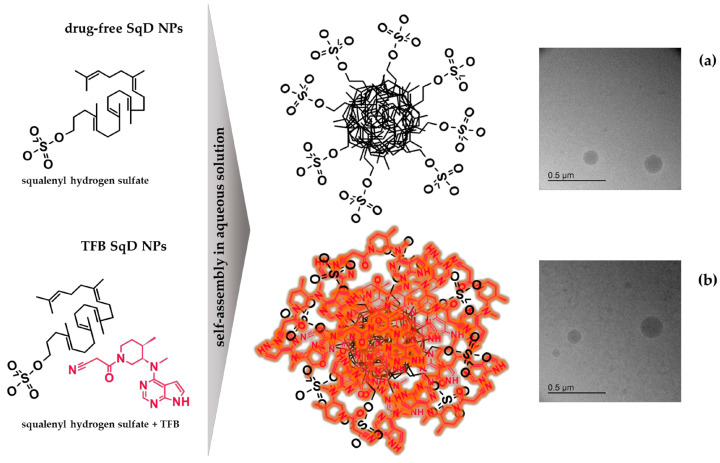
Schematic illustration of SqD and TFB chemical structures, and the self-assembled drug-free SqD NPs and TFB SqD NPs. Morphology of (**a**) drug-free SqD NPs and (**b**) TFB SqD NPs visualized by cryo-TEM. Black scale bar equals 0.5 μm.

TFB SqD NPs were prepared using free base TFB and anionic squalenyl derivative (SqD)—squalenyl hydrogen sulphate (Figure 1)—whose detailed synthesis and characterization can be found in our previous work [43,44]. The optimal TFB SqD NPs were produced by solvent evaporation technique. In detail, TFB and SqD were separately solubilized in THF at a concentration of 2 mg/mL and 10 mg/mL, respectively. An appropriate volume of each compound solution in THF was mixed to obtain a final TFB:SqD (36.5:63.5 *w*/*w*) and a total 1 mg/mL concentration. Subsequent, phosphate buffer (10 mM) pH 6.75 (phos-buffer) was added reaching a THF:phos-buffer volume ratio of 1:0.9. The TFB SqD NPs were formed upon slow evaporation of THF, and water was then partially removed (at 40 mbar, 40 °C, in a Rotavapor R-300, Büchi, Essen, Germany) to reach a final TFB SqD NPs concentration of 5.5 mg/mL, which contained an equivalent TFB concentration of 2 mg/mL. Drug-free SqD NPs were prepared using the same procedure and served as controls.

### 2.4. Quantification of Drug Loading Capacity and Encapsulation Efficiency

The quantity of drug in the TFB SqD NPs was determined using a direct method. In brief, 500 μL of the TFB SqD NPs 5.5 mg/mL suspension was centrifuged at 24.400 g, 4 °C, for 4 h. Both supernatant and NPs sediment were collected, of which the supernatant was further diluted in MeOH and ready for high performance liquid chromatography (HPLC, Supplemental part 2) analysis, while the NPs sediment was freeze dried (Lyo-Cube 4-8, Alpha 2-4 LSC, Christ, Osterrode am Harz, Germany). Following the total weight measurement of the dried NPs sediment, it was solubilized in a predetermined volume of MeOH, then sonicated for 1 h in an ultrasound bath (Bandelin, Sonorex, Bandelin electronic GmbH and Co. KG, Berlin, Germany). The subsequent solution was diluted in MeOH and analyzed by HPLC (Supplemental part 2). The encapsulation efficiency (EE) and the drug loading capacity (LC) of TFB SqD NPs were calculated as follows:

EE (%) = (TFB amount in NPs sediment/total TFB amount) × 100(1)

LC (%) = (TFB amount in NPs sediment/total weight of NPs sediment) × 100(2)

where in:

total TFB amount = TFB amount in the NPs sediment + TFB amount in the supernatant(3)

At least three experiments were conducted and results are presented as mean ± standard deviation (SD).

### 2.5. Size, Polydispersity Index, Zeta-Potential, and pH Value Determination

The hydrodynamic diameter (reported as intensity-based z-average) and the polydispersity index (PdI) were determined by dynamic light scattering (DLS) using a Zetasizer (Zetasizer Nano ZSP, ZEN5600, Malvern, UK, Software 7.02). The zeta-potential was determined by electrophoretic light scattering (ELS), using the same equipment. The Zetasizer was equipped with a He-Ne Laser at a wavelength of 633 nm, DLS was done at a backscattering angle of 173° and ELS at an angle of 13°. For the measurements, 20 μL of the final NP-suspension was diluted in 800 μL water. The pH of the final NP-suspensions and aqueous drug solution of TFB-C in phos-buffer (2 mg TFB/mL) (aqTFB-C) was determined by a pH meter (Mettler Toledo Seven Compact PHS 210 including pH electrode Mettler Toledo InLab^®^ Micro, Switzerland). To determine the colloidal storage stability of TFB SqD NPs and drug-free SqD NPs at 4 °C, the measurements were repeated after 12 and 24 days. Three experiments were conducted and measurements were done in triplicates. Results are reported as mean ± SD.

### 2.6. Morphology

The morphology of the TFB SqD NPs and of the drug-free SqD NPs were determined by cryogenic transmission electron microscopy (cryo-TEM). Three microliters of each NP-suspension were plotted for two seconds on a holey carbon grid (S147-4, Plano, Wetzlar, Germany) and subsequently plunged into −165 °C liquid ethane. Under liquid nitrogen conditions they were transferred to a Gatan model 914 cryo-TEM sample holder (Pleasonton, CA, USA). The sample analysis was done at −170 °C under low dose conditions, using a JEOL (Akishima, Tokio, Japan) JEM-2100 LaB6 TEM equipped with a Gatan Orius SC1000 CCD camera to gain bright field images.

### 2.7. In Vitro Release Study

The *in vitro* release profile of TFB SqD NPs was determined at pH 5.0 (ammonium acetate buffer, 10 mM) and 32 °C to simulate skin conditions [48], over a 24 h period using a dialysis bag method. In brief, following the equilibration of the dialysis membrane in the ammonium acetate buffer, 900 μL of the final TFB SqD NPs suspension was loaded into a dialysis bag consisting of regenerative cellulose (MWCO 10 kDa). Subsequently, the loaded dialysis bag was sunk in 30.0 mL ammonium acetate buffer, and the whole system was shaken at 100 rpm, 32 °C. At predetermined time points (0.25, 0.5, 0.75, 1, 2, 4, 6, and 24 h), 500 μL of the release medium was withdrawn, and an equal volume of fresh buffer was added. The samples were then analyzed by HPLC (Supplemental part 2). The maximum solubility of TFB in the ammonium acetate buffer was determined at 115.6 ± 9.3 μg/mL prior to the experiment. Three experiments were conducted and results are expressed as mean ± SD.

### 2.8. Ex Vivo and In Vivo Performance of TFB SqD NPs

We evaluated the HFs targeted transport and therapeutic efficacy of TFB SqD NPs in comparison to plain drug solutions in an *ex vivo* pig ear model and an allergic dermatitis mouse model, respectively.

#### 2.8.1. Targeted Follicular Transport of TFB in a Pig Ear Model

Excised hairy human skin cannot be used for follicular penetration studies of therapeutics, due to the shrinkage of the follicular reservoir of around 90%. This effect can be precluded in the pig ear model, where the skin remains on the cartilage [49,50,51]. Hence, in this study, we investigated the follicular and interfollicular penetration and permeation of TFB (the terms penetration and permeation are defined in the Supplemental part 4 and used according to Ref. [52]) in a pig ear model, which we optimized from two previously described methods, namely the differential stripping method [45,53] and the single HF harvesting method [46]. The differential stripping consists of two major steps—the surface cleaning by tape stripping to remove the formulation left on the skin surface, and the harvesting of the follicular casts by cyanoacrylate biopsies, including the formulation deposited in HFs. Unfortunately, the cyanoacrylate biopsies were not compatible and interfered with the TFB LC-MS quantification method. Therefore, the harvesting of the HFs was done as previously described by Kalia and coworkers: intact HFs were collected by 1 mm diameter punch biopsies (follicular skin). Accordingly, interfollicular drug penetration was determined by HF-free 1 mm diameter punch biopsies (interfollicular skin) [46]. The investigated drug formulations and tested vehicle control groups are summarized in Table 1.

**Scheme 1:**
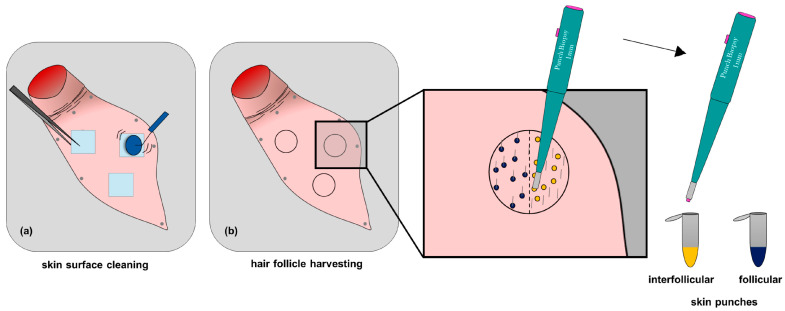
Graphic illustration: targeted follicular transport study of TFB in a pig ear model (**a**) skin surface cleaning by means of tape stripping and (**b**) follicular and interfollicular skin harvesting by 0.785 mm^2^ punch biopsies by means of stereomicroscopy.

The experiments on the pig ear model were as follows: the ears were collected from freshly slaughtered pigs, cleaned with water, and carefully dried with paper tissues. The ears were stored for a maximum of 7 days at 4 °C. Only ears with intact skin were used and the experiments were conducted on the outer auricle of the ear. In an experiment, a pig ear was firstly fixed on an aluminum foil covered wooden board. Subsequently, the hair shafts were shortened to 1 mm with an electrical clipper, during which the skin barrier was surely maintained (Braun Bartschneider, BT3040, Procter and Gamble Service GmbH, Schwalbach am Taunus, Germany). The areas for investigation with a surface area of 1.767 cm^2^ were marked, and there were at least two areas for the treatment group and one area for the vehicle control on each ear (Table 1). A 15 μL of the formulation—treatment or vehicle control—was applied on each marked area, which was then massaged with a gloved fingertip for 3 min to enhance the penetration into HFs [54]. After 1 h incubation at 32 °C, the skin surface was cleaned by means of tape stripping (Scheme 1a) [45,53].

For sufficient skin surface cleaning, 10 subsequent tape strips were pressed onto the skin surface by using a paint roller to minimize the influence of furrows and wrinkles [45,55]. Afterwards, the 1 mm diameter punch biopsies (equals 0.785 mm^2^) of follicular and interfollicular skin were collected as described in Scheme 1b. Briefly, the surface-cleaned area of investigation was equally divided into two parts. On one part, all HFs—described in Scheme 1b follicular skin, dark blue punches—were collected by means of a stereomicroscope (Model SZX7, SZX2-ILLTQ, Olympus Corporation, Tokyo, Japan). On the other part, the interfollicular skin—described in Scheme 1b interfollicular skin, orange punches—were collected. Notably, an equal number of punch biopsies were collected in each part and stored in 2 mL tubes. Moreover, a skin area of 4.91 cm^2^ including the rest of the treated area and the surrounding skin was also punched (called “skin rest”) for TFB mass balance calculation purposes [45]. Furthermore, TFB left on the materials that were in contact with the formulation, including glove-fingertip, tape strips, and skin rest, were extracted in 2 mL of a mixed MeOH:ammonium acetate buffer (pH 5.0, 5 mM) ratio of 60:40 *v/v* (called “extraction media”). The 0.785 mm^2^ punch biopsies were extracted in 500 μL extraction media. All samples were sonicated in an ultrasonic bath (Sonorex, Bandelin electronic GmbH and Co. KG, Berlin, Germany) for 1 h, subsequent shaking at 350 rpm for 2 h. Following, the samples were centrifuged at 24.400 g, at 20 °C for 5 min, the supernatant was filtered and if necessary diluted prior to LC-MS analysis (Supplemental part 3). According the FDA bioanalytical method validation guideline sensitivity criteria [56], the lower limit of quantification (LLOQ) was at least 5 times higher than the response from the blank matrix value. The LLOQ was determined separately for each different matrix type, by multiplying the highest found blank matrix value times 5, and the values below LLOQ were excluded. The TFB mass balance was determined for each treated area as follows:

Mass balance (%) = ((TFB in glove-fingertip + TFB in tape strips + TFB in follicular and interfollicular skin punches + TFB in skin rest)/applied TFB amount on treated area) × 100(4)

Results are reported as mean ± SD, illustrated by a scatter dot plot.

#### 2.8.2. The Therapeutic Efficacy of TFB in an Allergic Dermatitis Mouse Model

In this study, we employed the allergic dermatitis mouse model to gain the first insights in the therapeutic efficacy of the TFB SqD NPs. The efficacy of the TFB SqD NPs in reducing inflammatory response was compared to the positive control—TFB-acet:DMSO—and aqTFB-C formulation (Table 2). Drug-free SqD NPs and acetone:DMSO solution served as vehicle control groups. The reduction of the ear swelling at 24 h post-challenging by allergen served as readout to evaluate the efficacy of a formulation in reducing inflammatory response in mice.

**Scheme 2:**
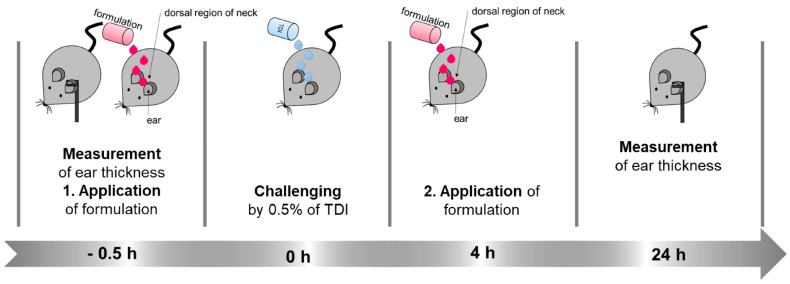
Graphic illustration of the chronology of the formulation application, ear thickness measurements, and challenging time points of the allergic dermatitis mouse model. Each group contained of 7 female BALB/c (BALB/cAnNCrl) mice. The ability of the applied formulations to reduce inflammatory response was monitored by ear swelling reduction 24 h post-allergen challenge (TDI: toluene 2,4 diisocyanate).

The animal procedure was as follows: prior to the experiments, the allergic dermatitis mouse model was established by sensitizing and challenging the mice for one month with the contact allergen toluene 2,4 diisocyanate (TDI) in acetone as described in previous studies [47,57,58]. On the experiment day, the ear thickness was measured and the formulations were applied (Scheme 2, −0.5 h). After 30 min, the mice were challenged by applying 20 μL and 30 μL of 0.5% TDI in acetone on the mouse ear and the neck’s dorsal region, respectively (Scheme 2, 0 h). After 4 h, the formulations were applied for the second time (Scheme 2, 4 h). The ear thickness was measured again at 24 h post-challenge, and the mice were sacrificed afterwards (Scheme 2, 24 h). The reduction of the ear swelling was determined by subtracting the ear thickness after 24 h of challenge from the ear thickness before the challenge. Results are reported as mean ± standard error (SEM).

### 2.9. Statistics

Statistical analysis was performed by GraphPad Prism 8.0 or Microsoft Excel 2016/2019. Significant differences between formulations in the *ex vivo* pig ear experiment were tested for follicular and interfollicular skin area separately by one-way ANOVA with multiple comparisons by Tukey’s test. Significant difference between the vehicle control group and treatment group in *in vivo* mouse model was detected by an unpaired two-tailed *t*-test. Significance levels are reported as follows: *p* ≥ 0.05: n.s., *p* = 0.01 to 0.05: *, *p* = 0.001 to 0.01: **, and *p* < 0.001: ***. 

## 3. Results and Discussion

### 3.1. Characteristics of TFB SqD NPs

The stable and uniform TFB SqD NPs with a size of 239.1 ± 43.5 nm and PdI of 0.21 ± 0.01 (Table 3 and Figure S1) were formed by simultaneous self-assembly of the amphiphilic SqD and TFB using the solvent evaporation method. The anionic SqD (Figure 1) containing a hydrophilic hydrogen sulfate head-group and a hydrophobic squalenyl moiety potentially allows the loading of TFB (Figure 1) in both compartments via charged and hydrophobic interactions, respectively.

We have previously reported the significant drug LC of SqD NPs yet also pointed out that the loading of small molecules—containing polar groups and having rather poor water-solubility—is challenging, only allowing e.g., the maximum LC at around 10% of a quorum sensing inhibitor [43] or at around 9% of Nile red [44]. In this study, it is surprising that the LC of TFB could be maximized at 20.1% ± 2.2%, with a corresponding EE at 19.8% ± 3.6%. Most importantly, such a high LC of TFB in SqD NPs enhanced the TFB solubility in phos-buffer up to 2 mg TFB/mL, which was around 40 times higher than the maximum solubility of TFB alone in the same buffer (47.9 ± 0.2 μg/mL). The negative zeta-potential of the drug-free SqD NPs (−54.5 ± 1.7 mV, Table 3) did not change upon drug loading (−59.9 ± 2.3 mV, Table 3), which led to the assumption that TFB might be loaded rather via hydrophobic than charged interactions into SqD NPs [44]. It is worth noting that the pH value plays a crucial role in the optimization of TFB loading. As the pH of human skin is slightly acidic around 5 [59], a comparable pH of the formulation is hence preferred. However, the better solubility of TFB in acidic pH [60] does not benefit the NP encapsulation. In our optimization, an ideal compromise was found by preparing the TFB SqD NPs in a phos-buffer, resulting in a suspension with a pH of 6.3 (Table 3).

The low negative zeta potential of the NPs (−59.9 ± 2.3 mV, Table 3) could stabilize the suspension well by strong charge repulsion [61]. The drug-free SqD NPs having similar characteristics to the TFB SqD NPs—a hydrodynamic size of 225.7 ± 20.3 nm, PdI of 0.19 ± 0.02, and zeta-potential of −54.5 ± 1.7 mV—served as a control (Table 3 and Figure S2). The morphology of the NP-suspensions was visualized by cryo-TEM, demonstrating homogeneous, dense, and spherical shaped NPs in both drug-free SqD NPs (Figure 1a) and TFB SqD NPs (Figure 1b).

### 3.2. Stability of TFB SqD NPs

**Figure 2:**
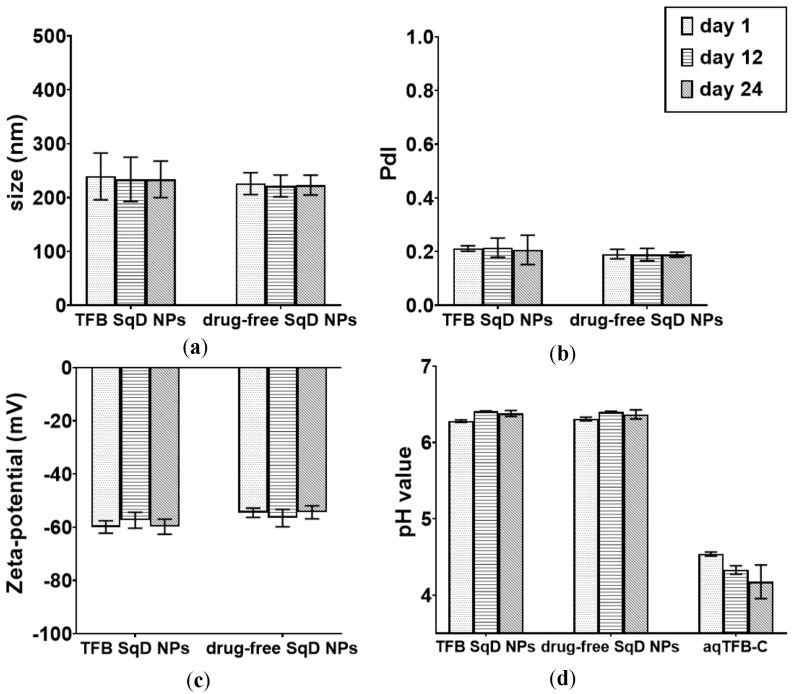
Colloidal stability of TFB SqD NPs, drug-free SqD NPs, and aqTFB-C solution at 4 °C, over a 24-day period, investigated by DLS, ELS, and pH measurements. (**a**) Hydrodynamic diameter (nm), (**b**) polydispersity index, (**c**) zeta-potential (mV), and (**d**) pH-value. Results are presented as mean ± SD.

Both drug-free SqD NPs and TFB SqD NPs exhibited good colloidal stability at storage conditions over 24 days (Figure 2a–c). Interestingly, the aqTFB-C solution (2 mg TFB/mL) in phos-buffer, showed a lower pH value at 4.54 ± 0.03 compared to the NP formulations and was further decreasing to 4.17 ± 0.22 after 24 days at 4 °C (Figure 2d). This can be explained by the interaction of the citrate salt with the disodium hydrogen phosphate, which forms a new buffer system and lowers pH [62]. Notably, crystals were visible in the TFB-C in phos-buffer solution upon storage at 4 °C after 2 weeks. No visual changes were observed over the 24-days in drug-free SqD NPs and TFB SqD NPs. 

### 3.3. Release Profile of TFB SqD NPs

**Figure 3:**
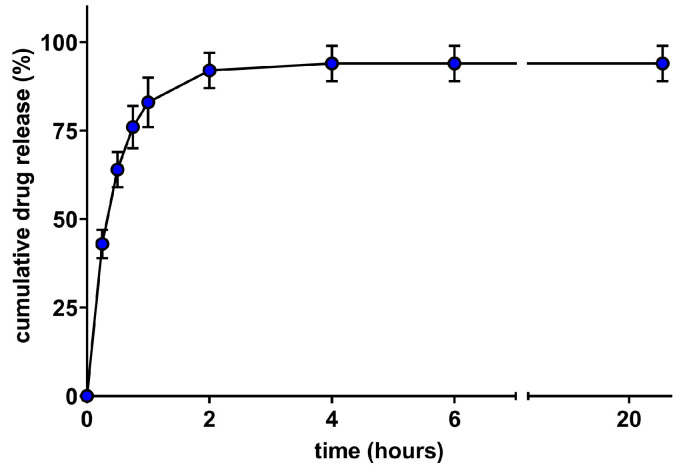
*In vitro* cumulative drug release study of TFB SqD NPs at pH 5.0 and 32 °C over 24 h. Results are presented as mean ± SD.

We evaluated the *in vitro* release profile of TFB SqD NPs and accessed the TFB stability in skin mimicking conditions using HPLC [48,59]. Figure 3 shows a burst TFB release of around 40% in the first 0.25 h followed by a continuous slower release of TFB until complete. This data set proves the releasing capacity of the TFB SqD NPs. Moreover, the HPLC analysis of TFB over 24 h did not show any signs of drug degradation. It is also important to highlight that a simple correlation between *in vitro* and *in vivo* release of TFB SqD NPs cannot be drawn, since the lipid derived SqD NPs would interact differently with the sebum milieu inside the HFs. In addition, the changing pH profile inside HFs was not taken into account [63,64]. Therefore, further *in vivo* experiments would be necessary to evaluate the release profile of TFB SqD NPs in HFs.

### 3.4. Ex vivo and In vivo Performance of TFB SqD NPs

#### 3.4.1. Targeted Follicular Transport of TFB in a Pig Ear Model

**Figure 4:**
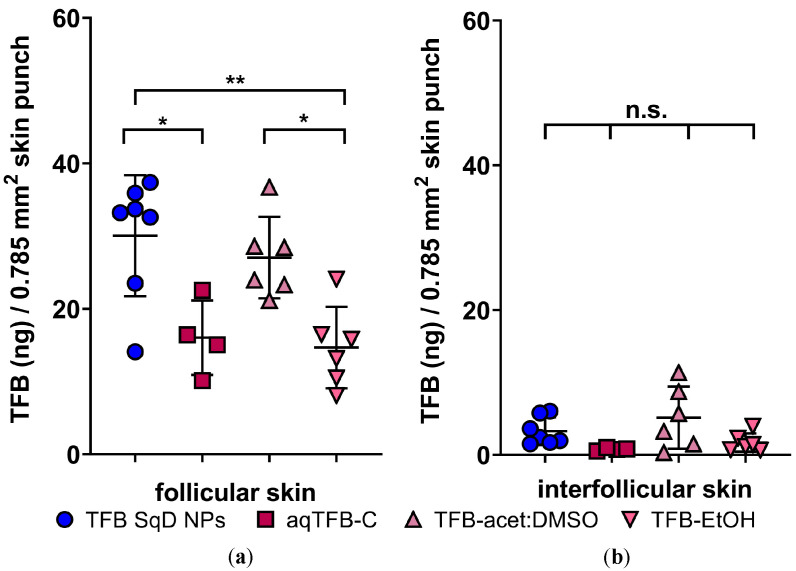
TFB deposition in nanograms per 0.785 mm^2^ skin punch of surface-cleaned application area in (**a**) follicular skin and (**b**) interfollicular skin. Treatment groups are presented as TFB SqD NPs in blue circles, aqTFB-C in dark red squares, TFB-acet:DMSO in purple upward triangles, and TFB-EtOH in pink downward triangles respectively. Results presented as mean ± SD in a scatter dot plot. Significant differences are presented as *p* ≥ 0.05: n.s., *p* = 0.01 to 0.05: *, and *p* = 0.001 to 0.01: **, tested for follicular and interfollicular skin separately by one-way ANOVA with multiple comparisons by a Tukey’s test.

We first investigated the targeted follicular delivery of TFB in a pig ear model. The quantity of TFB in nanograms per 0.785 mm^2^ follicular (Figure 4a) and interfollicular (Figure 4b) skin surface cleaned punch biopsy was analyzed. The results were used to compare the performance of TFB SqD NPs to TFB alone formulations—aqTFB-C, TFB-EtOH, and TFB-acet:DMSO. Moreover, TFB-acet:DMSO has shown a significant effect in an allergic dermatitis mouse model compared to vehicle control [47]. Therefore, it was used as a standard to compare the performance of TFB SqD NPs.

Overall, the results clearly demonstrated that the follicular transport was the major route for drug penetration compared to the interfollicular route. Figure 4a compares the TFB amount in the follicular skin delivered by different formulations. The aqTFB-C and TFB-EtOH delivered 16.03 ± 5.11 ng/0.785 mm^2^ and 14.68 ± 5.59 ng/0.785 mm^2^ of TFB, respectively, into the follicular skin, which were significantly less than that of the TFB SqD NPs at 30.06 ± 8.32 ng/0.785 mm^2^. Interestingly, the ability of SqD NPs to deliver TFB to the follicular skin was found to be as good as the TFB-acet:DMSO, which allowed the TFB delivery of 27.03 ± 5.60 ng/0.785 mm^2^.

As shown in Figure 4b, the TFB amount in interfollicular skin was, in contrast, relatively low and not significantly different regardless of different tested formulations. The amount of TFB penetrating interfollicular skin was slightly more pronounced when using the TFB-acet:DMSO (5.14 ± 4.29 ng/0.785 mm^2^, Figure 4b) compared to the other tested formulations (3.28 ± 1.91 ng/0.785 mm^2^ for TFB SqD NPs, 0.77 ± 0.20 ng/0.785 mm^2^ for aqTFB-C, and 1.70 ± 1.26 ng/0.785 mm^2^ for TFB-EtOH, Figure 4b). The overall higher drug penetration by follicular skin compared to interfollicular skin stresses the impact HFs have on general drug penetration into skin, and are in good agreement to previous findings [65].

The guideline of the scientific committee on consumer safety recommends a mass balance in the range of 100% ± 15% [66], these criteria were met for all formulation types except for the TFB-acet:DMSO (Table 4). In addition to the total organic solvent-based formulation of TFB-acet:DMSO, the lack of mass recovery leads to the assumption of uncontrolled and fast drug penetration and permeation. Taking together, these results imply the advantages of using aqueous SqD NPs for the targeted follicular delivery of TFB.

#### 3.4.2. The Therapeutic Efficacy of TFB in an Allergic Dermatitis Mouse Model

The topical formulation of TFB in an organic mixture of acetone:DMSO (7:1 *v*/*v*) has previously shown a reduction in inflammatory response in an allergic dermatitis mouse model at all tested TFB concentrations that ranged from 0.1% to 0.5% (*w*/*v*) compared to vehicle controls [47]. These results implied the therapeutic efficacy of TFB at preclinical levels, and validated the allergic dermatitis mouse model [47]. This Th2 driven mouse model resembles major characteristics of acute atopic dermatitis (AD) lesions in regard to cytokine pattern and prediction of effectiveness of, e.g., phosphodiesterase 4 inhibitors [67], JAK-inhibitors [47], and glucocorticoids [57], all therapeutic options used or in clinical phase for treatment of human AD.

**Figure 5:**
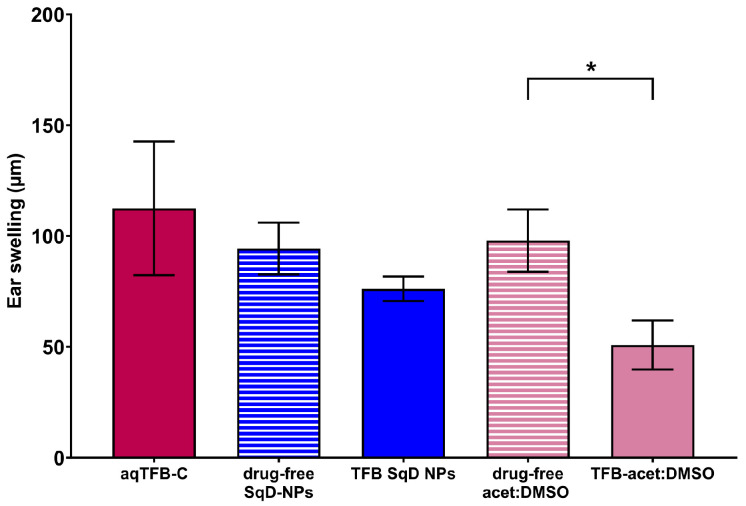
Ear swelling reduction in *in vivo* allergic dermatitis mouse model after 24 h allergen challenge. Application of formulation 30 min prior and 4 h after challenging. Tested treatment groups are: aqTFB-C (filled dark red bar), TFB SqD NPs (filled dark blue bar), including control group drug-free SqD NPs (striped dark blue bar), and TFB-acet:DMSO (filled purple bar), including control group drug-free acet:DMSO (striped purple bar). Results are presented as mean ± SEM. Significant difference between vehicle control group and treatment group was tested by an unpaired two-tailed *t*-test. Significance levels are reported as *p* ≥ 0.05: n.s. and *p* = 0.01 to 0.05: *.

Therefore, we used this model and TFB-acet:DMSO as a positive treatment control to evaluate the therapeutic efficacy of TFB SqD NPs and aqTFB-C. Notably, it should be taken into account that the acetone:DMSO solution cannot be considered as a safe drug delivery system for topical application. DMSO itself might alter the cellular processes and cause some unknown biological effects [68,69]. However, DMSO containing formulations are often used for preclinical research due to good drug solubility in DMSO and penetration enhancing effects [69]. As shown in Figure 5, mice treated with TFB-acet:DMSO demonstrated a significant reduction of around 50% in ear swelling compared to that from the drug-free acet:DMSO (50.80 ± 11.09 μm and 97.97 ± 14.05 μm ear swelling, respectively), which is in agreement with Fukuyama et al. [47]. The TFB SqD NPs were able to reduce the ear swelling by around 20% compared to drug-free SqD NPs (76.20 ± 5.54 μm and 94.34 ± 11.71 μm ear swelling, respectively). The aqTFB-C did not show any reducing effect (112.5 ± 30.16 μm ear swelling).

In general, these results were actually well correlated to the *ex vivo* pig ear outcomes. Particularly, the aqTFB-C provided a very low penetration and permeation in both follicular and interfollicular skin *ex vivo*, also performed poorly in the allergic dermatitis mouse model *in vivo*. Despite resulting a comparable TFB delivery in follicular skin in the pig ear model, TFB SqD NPs did not reduce the inflammatory response as much as TFB-acet:DMSO did in the mouse model. Such results could be explained by the higher TFB interfollicular penetration and permeation of TFB-acet:DMSO, and it has to be taken into account, that not only the follicular skin, but the whole treated skin area (follicular and interfollicular) was affected in the allergic dermatitis mice model. Furthermore, the low mass balance of TFB-acet:DMSO in the pig ear model also suggested that this formulation had uncontrollable penetration and permeation rates and depth into the skin, possibly leading to an overall higher delivered TFB amount into the whole skin tissue. As also discussed in the previous study, the addition of DMSO as a vehicle for NP-suspension is likely to enhance the interfollicular skin permeation, while the aqueous NP-suspension only maximized the follicular deposition [70]. These results demonstrate quite plainly that it is important to take *ex vivo* data of penetration and permeation pathways into account when evaluating *in vivo* data of drug effects of topically applied formulations. Taking the shown follicular targeting of the *ex vivo* pig ear model and the measurable anti-inflammatory effect of the *in vivo* mouse model into consideration, our TFB SqD NP was clearly the best option for safe and efficient delivery of TFB into the HF. The appropriate TFB concentration for a topical delivery and the overall biological effects have to be further determined, to meet the needs for a safe and effective nanomedicine against AA and other skin diseases.

## 4. Conclusions

In this study, we successfully showed the preparation of TFB SqD NPs with a satisfying drug LC of 20% and an enhanced drug solubility, up to 40 times in phos-buffer compared to the plain drug. Moreover, we maximized the targeted delivery of TFB into the hair follicles, as demonstrated in an *ex vivo* pig ear model. Thereby, NPs were able to provide controlled drug delivery to the target site, which is expected to minimize adverse effects due to high drug amounts in the interfollicular area and high systemic drug levels. While safe and well tolerated by avoiding any organic solvent, TFB SqD NPs showed a pharmacological effect in an *in vivo* mouse dermatitis model. Taking the *ex vivo* and *in vivo* results together, we have clearly demonstrated, that TFB SqD NPs are favorable to achieve controlled and targeted delivery of TFB into HFs.

## Figures and Tables

**Table 1:** Abbreviation, composition, formulation type, number of pig ears, and number of application areas used for *ex vivo* pig ear study on topical penetration and permeation pathways.

Group	Abbreviation	Components	Formulation Type	No. Ear	No. Area
Treatment (TFB 2 mg/mL)	TFB SqD NPs	TFB ^1^, SqD ^2^, phos-buffer ^3^	suspension	4	7
	aqTFB-C	TFB-C ^4^, phos-buffer ^3^	solution	2	4
	TFB-EtOH	TFB ^1^, EtOH ^5^:H_2_O (50:50 *v*/*v*)	solution	3	6
	TFB-acet:DMSO	TFB ^1^, acetone:DMSO ^6^ (7:1 *v*/*v*)	solution	3	6
vehicle control	phos-buffer	phos-buffer ^3^	solution	4	4
	EtOH:H_2_O	EtOH ^5^:H_2_O (50:50 *v*/*v*)	solution	3	3
	acet:DMSO	acetone:DMSO ^6^ (7:1 *v*/*v*)	solution	3	3

^1^ tofacitinib free base, ^2^ anionic squalenyl derivative, ^3^ phosphate buffer, ^4^ tofacitinib citrate, ^5^ ethanol, ^6^ dimethyl sulfoxide.

**Table 2:** Abbreviation, composition, formulation type, and number of treated mice of treatment and respective vehicle control groups for *in vivo* dermatitis mouse model study on biological response upon topical application.

Group	Abbreviation	Components	Formulation Type	No. Mice
Treatment TFB 2 mg/mL	TFB SqD NPs	TFB ^1^, SqD ^2^, phos-buffer ^3^	suspension	7
	aqTFB-C	TFB-C ^4^, phos-buffer ^3^	solution	7
	TFB-acet:DMSO	TFB ^1^, acetone:DMSO ^5^ (7:1 *v*/*v*)	solution	7
vehicle control	Drug-free SqD NPs	SqD ^2^, phos-buffer ^3^	suspension	7
	Drug-free acet:DMSO	acetone:DMSO ^5^ (7:1 *v*/*v*)	solution	7

^1^ tofacitinib free base, ^2^ anionic squalenyl derivative, ^3^ phosphate buffer, ^4^ tofacitinib citrate, ^5^ dimethyl sulfoxide.

**Table 3:** Physicochemical characteristics, including size, polydispersity index (PdI), zeta-potential, pH, loading capacity (LC) and encapsulation efficiency (EE), of SqD nanoparticles (NPs) determined by dynamic light scattering (DLS), electrophoretic light scattering (ELS), pH measurements, and HPLC. Results are reported as mean ± SD.

Physicochemical Characteristic	Drug-Free SqD NPs	TFB SqD NPs
size (nm)	225.7 ± 20.3	239.1 ± 43.5
PdI	0.19 ± 0.02	0.21 ± 0.01
zeta-potential (mV)	−54.5 ± 1.7	−59.9 ± 2.3
pH	6.28 ± 0.02	6.31 ± 0.02
LC (%)	N.A.	20.1 ± 2.1
EE (%)	N.A.	19.8 ± 3.6

**Table 4:** Mass balance of TFB applied on application area in *ex vivo* pig ear skin experiment. Results are presented as mean ± SD.

Treatment Group	Mass Balance (%)
TFB SqD NPs	90.4 ± 6.4
aqTFB-C	87.4 ± 1.9
TFB-acet:DMSO	69.7 ± 10.7
TFB-EtOH	95.7 ± 3.3

## Supplementary Materials

The following are available online at https://www.mdpi.com/1999-4923/12/12/1131/s1, Part 1, Particle size distribution data by dynamic light scattering (DLS) of SqD NPs, Figure S1: Particle size distribution of TFB SqD NPs determined by DLS, Figure S2: Particle size distribution of drug-free SqD NPs determined by DLS; Part 2, High performance liquid chromatography method used for quantitative tofacitinib (TFB) determination; Part 3, LC-MS/MS method used for quantitative tofacitinib (TFB) determination; part 4, Definitions.
