# Supplementary material for: Tofacitinib Loaded Squalenyl Nanoparticles for Targeted Follicular Delivery in Inflammatory Skin Diseases
Source: Pharmaceutics. 2020 Nov 24;12(12):1131. doi: 10.3390/pharmaceutics12121131 (PMC7760822; doi:10.3390/pharmaceutics12121131)
Supplement: Supplementary file 1 [file pharmaceutics-12-01131-s001.pdf]

pharmaceutics

Supplementary Materials: Tofacitinib Loaded Squalenyl Nanoparticles for Targeted Follicular Delivery in Inflammatory Skin Diseases

Rebekka Christmann, Duy-Khiet Ho, Jenny Wilzopolski, Sangeun Lee, Marcus Koch, Brigitta Loretz, Thomas Vogt, Wolfgang Bäumer, Ulrich F. Schaefer, Claus-Michael Lehr *

1. Particle Size Distribution Data by Dynamic Light Scattering (DLS) of SqD NPs

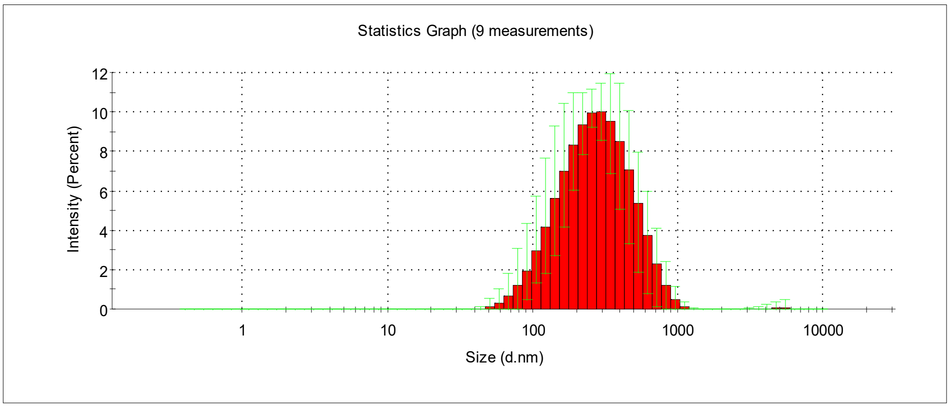

Figure S1. Particle size distribution of TFB SqD NPs determined by DLS. Results are presented as mean ± SD, *n*=3, and measurement are done in triplicate (Zetasizer Nano ZSP, ZEN5600, Malvern, UK, Software 7.02).

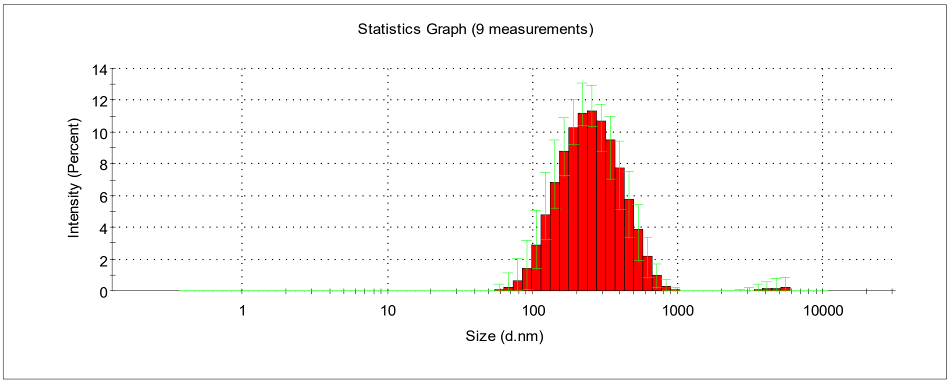

Figure S2. Particle size distribution of drug-free SqD NPs determined by DLS. Results are presented as mean ± SD, *n* = 3, and measurements are done in triplicate (Zetasizer Nano ZSP, ZEN5600, Malvern, UK, software 7.02).

2. High Performance Liquid Chromatography Method used for Quantitative Tofacitinib (TFB) Determination

The quantitative HPLC analysis of TFB was modified after Ref. [1]. A Dionex UltiMate 3000 system including LPG-3400SD pump, WPS-3000 auto sampler, TCC-3000 column oven and a DAD3000 detector were used (Thermo-Fischer Scientific, Dreieich, Germany). The sample injection volume was 10 µL. The analysis was done by an isocratic method with a flow rate at 200 µL/min, a mobile phase consisting of 45% MeOH, 55% pH 5.0 buffer (ammonium acetate, 10 mM) using a Syncronis C18 column (150 mm × 2.1 mm, 3 µm particle size, Thermo Scientific, Dreieich, Germany) at 35 °C. TFB was detected at 6.1 minutes retention time and 287 nm wavelength. The method was linear over a range of 0.25–10 µg/mL (r^2 = 0.9999). Lower limit of quantification (LLOQ) and lower

limit of detection (LLOD) were determined experimentally: LLOQ 0.25 µg/mL and LLOD 0.06 µg/mL, respectively.

Calculations and analysis were done by Chromeleon 7 (Thermo Scientific Dionex, Dreieich, Germany), Microsoft Excel 2016/2019, or GraphPad Prism 8.0.

3. LC–MS/MS Method used for Quantitative Tofacitinib (TFB) Determination

The quantitative TFB determination in ex vivo pig ear experiment was done by LC–MS/MS, modified after Ref. [1,2]. An Accela UHPLC system was coupled to a TSQ Quantum Access Max. The software used to operate the system were Thermo TSQ-Tune master and Thermo Xcalibur. An Accucore RP-MS column (150 mm × 2.1 mm, 2.6 µm particle size, Thermo Fisher Scientific, Waltham, MA, US) was used. An isocratic method was run at a flow of 200 µL/min of 45% MeOH + 0.1% formic acid and 55% pH 5.0 (ammonium acetate buffer, 5 mM) for 6 minutes, the retention time of TFB was around 3 minutes. The sample injection volume was 10 µL, the controlled tray temperature was 4 °C and the column oven temperature 35 °C. For detection, heated electrospray ionization (H-ESI) in the positive mode was used with the best conditions: spray voltage at positive polarity 3500 V, capillary temperature 300 °C, vaporizer temperature 320 °C, auxiliary gas (nitrogen) pressure 10 arbitrary units, and sheath gas (nitrogen) pressure 50 arbitrary units. The selective reaction monitoring (SRM) was chosen for quantification and ions were observed as follows: parent ion at 313.132 (*m*/*z*); collision energy 28 V, product ion at 149.129 (*m*/*z*). Samples were diluted prior LC-MS/MS analytic accordingly and the method was validated over four calibration ranges 2.5–50 ng/mL (r^2 = 0.9986), 30–200 ng/mL (r^2 = 0.9972), 150–500 ng/mL (r^2 = 0.9961), and 400–850 ng/mL (r^2 = 0.9824). The lower limit of quantification (LLOQ) of the method was 2.5 ng/mL TFB.

The LLOQ was determined for each matrix and each blank formulation separately (matrix: tape strips, follicular punches, interfollicular punches, skin rest punch, glove fingertip and formulations: phosphate buffer (pH 6.75), EtOH:H_2O (50:50 *v*/*v*), and acetone:DMSO (7:1 *v*/*v*)), according to the FDA bioanalytical method validation guideline sensitivity criteria [3]: the LLOQ was at least 5 times higher than the response from the blank matrix value. The highest found blank matrix value was multiplied by 5, if the detected amount was not below the quantification limit of the method (2.5 ng/mL TFB). Matrix associated LLOQs for phosphate buffer could be calculated for tape strips 5 and 6, tape strips 7 and 8, and skin rest punch: 24.4, 12.4, and 40.3 ng/mL TFB, respectively.

For EtOH:H_2O (50:50 *v*/*v*) formulation matrix associated LLOQs were determined for skin rest punch with 13.4 ng/mL TFB and for acetone:DMSO (7:1 *v*/*v*) for follicular punches (34.8 ng/mL TFB) and skin rest punches (16.8 ng/mL TFB).

All samples were measured three times. Values below the LLOQ were excluded—no value had to be excluded.

4. Definitions

The terms penetration and permeation were defined and used according to Ref. [4]:

"Dermal penetration:	The movement of a chemical from the outer surface of the skin into the epidermis, but not necessarily into the circulatory system".
"Dermal permeation:	The penetration through one layer into another, which is both functionally and structurally different from the first layer".

References

1. Vijay, K.S.; Dhiman, V.; Giri, K.K.; Sharma, K.; Zainuddin, M.; Mullangi, R. Development and validation of a RP-HPLC method for the quantitation of tofacitinib in rat plasma and its application to a pharmacokinetic study. *Biomed. Chromatogr.* **2015**, *29*, 1325–1329, doi:10.1002/bmc.3426.
2. Sharma, K.; Giri, K.; Dhiman, V.; Dixit, A.; Zainuddin, M.; Mullangi, R. A validated LC-MS/MS assay for simultaneous quantification of methotrexate and tofacitinib in rat plasma: application to a pharmacokinetic study. *Biomed. Chromatogr.* **2015**, *29*, 722–732, doi:10.1002/bmc.3348.

3. FDA/CDER. Bioanalytical Method Validation Guidance for Industry. https://www.fda.gov/files/drugs/published/Bioanalytical-Method-Validation-Guidance-for-Industry.pdf (accessed on 7 August 2020).
4. Buist, H.; Craig, P.; Dewhurst, I.; Hougaard Bennekou, S.; Kneuer, C.; Machera, K.; Pieper, C.; Court Marques, D.; Guillot, G.; Ruffo, F.; et al. Guidance on dermal absorption. *EFSA J.* **2017**, *15*, e04873, doi:10.2903/j.efsa.2017.4873
